# The study on the response of erosion and deposition evolution in the main channel of the Tarim River to water and sediment conditions under different representative years

**DOI:** 10.1371/journal.pone.0334755

**Published:** 2025-10-23

**Authors:** Jiuzhou Gao, Lin Li, Quanli Zong, Wenhong Dai

**Affiliations:** 1 College of Water Resources and Civil Engineering, Xinjiang Agricultural University, Urumqi, China; 2 Xinjiang Key Laboratory of Water Resources Engineering Safety and Water Hazard Prevention and Control, Urumqi, China; 3 College of Resources and Environment, Qingdao Agricultural University, Qingdao, China; 4 College of Water Resources and Hydroelectricity, Hohai University, Nanjing, China; Universidade de Aveiro, PORTUGAL

## Abstract

To investigate the impact of different water and sediment conditions on the morphological shaping of the middle reaches of the Tarim River, this study establishes an erosion-deposition evolution model for the Yingbazha to Wusiman River section under the 2018 shoreline conditions using MIKE21 software and conducts validation. Five working conditions were selected for typical years of high-flow, normal-flow, and low-flow, as well as years of extreme floods and extreme droughts, to simulate the river channel’s erosion and deposition evolution under varying water and sediment conditions. By analyzing metrics such as erosion and deposition volume, depth of scour and fill, changes in the channel’s planform morphology, thalweg elevation variations, and cross-sectional changes along the river reach, the patterns of erosion and deposition evolution in this segment were systematically examined. The results indicate that: (1) Under different representative year conditions, the river was always in a net deposition state. The sediment deposition was highest in the extreme flood year (1.885 × 10^7^ tons, accounting for 34% of the incoming sediment) and lowest in the extreme drought year (1.109 × 10^6^ tons, accounting for 83% of the incoming sediment). The unit runoff sediment transport efficiency increased by 54% with the increase in flow. However, when the runoff exceeded the critical threshold of 3.7 × 10^9^ m^3^, the increase in scour volume (+440%) far outpaced the deposition volume (+143%), revealing a critical turning point in the erosion-deposition mechanism. (2) The erosion-deposition process follows a three-stage evolutionary pattern of “deposition-scouring-redeposition”: At low flow, insufficient sediment-carrying capacity leads to continuous deposition. After surpassing the critical flow, the sediment-carrying capacity dominates scouring. Under high flow conditions, the water and sediment volume increases sharply, restarting deposition. The deep pool elevation exhibits corresponding “rise-drop-rise” periodic fluctuations. (3) Through analysis of typical cross-sections, it is shown that in extreme flood years and typical wet years, the lateral swinging of the main channel causes scouring. However, the collapse of the bank and the widening of the river channel result in increased deposition in the main channel, leading to an overall elevation of the riverbed. In typical drought years and extreme drought years, due to lower flow, water levels, and flow velocities, the erosion-deposition process only occurs within the main river channel. The research results provide a better understanding of the erosion-deposition evolution trend of the meandering section of the Tarim River’s main channel, offering scientific guidance for the future development, management, and sustainable development of the middle reach of the Tarim River.

## 1. Introduction

The Tarim River, ranked as China’s largest and the world’s fifth-largest inland river, encompasses a drainage area of 1.02 × 10^6^ km^2^. Its main stem extends 1321 km, flowing eastward from Xiaojiake and subsequently southeastward into Taitema Lake, exhibiting characteristics of a plain-type river [1]. The mainstem of the Tarim River primarily exhibits three channel patterns: braided, transitional, and meandering. Specifically, the braided reach extends approximately 190 km from Xiaojiake downstream to the vicinity of Shaya County Deer Farm. The transitional reach spans between Xinqiman and Qumao Gejin, while the segment from Qumaogejin to Qiala constitutes the meandering reach [2]. The midstream reach of the Tarim River’s mainstem between Yingbazha and Wusiman is a characteristic meandering channel, flowing east-west with a total length of 179 km. This section exhibits frequent channel migration and substantial lateral displacement, reflecting highly active fluvial dynamics. The Yingbazha-Wusiman reach has been constructed with 28 ecological diversion sluices, one reservoir, and 207.34 km of embankments and revetments along its banks. The current riverside irrigation area covers approximately 9.34 × 10^4^ hm^2^. The Karquga Reservoir, with an effective storage capacity of 2.6 × 10^7^ m^3^, irrigates 1334 hm^2^, primarily supplying agricultural water for Karquga Township while also providing ecological water provision [3]. These anthropogenic activities inevitably alter the natural evolutionary trajectory of river channels, while the resultant human-modified fluvial processes reciprocally impact subsequent interventions [4–7]. Consequently, a mechanistic understanding of erosion-deposition dynamics in this reach is critical for optimizing dual objectives: ensuring flood-resilient infrastructure along both banks and guiding sustainable shoreline utilization strategies.

To systematically analyze the evolutionary trajectory of the Tarim River’s mainstem under coupled natural-anthropogenic influences, previous studies have conducted comprehensive investigations employing a suite of methodologies including satellite imagery interpretation, hydrological data analytics, and process-based numerical modeling. Scholars such as Gao Jiuzhou [8], Wang Xinmiao [9], Yu [10], and Li [11] have primarily employed remote sensing image interpretation and related methodologies to investigate the impacts of human activities, natural drivers, and ecological dynamics on river channel evolution within the Tarim River Basin. Research indicates that channel morphology exhibits distinct migration rates and variation patterns across different reaches, underscoring the urgent need for strengthened ecological remediation and conservation measures within the basin. Scholars including Zong [12], Zhang [13], Qi Hongkun [14], He Xinghong [15], and Wang Guangyan [16] have systematically analyzed hydrological, sediment, vegetation, and climatic data from the Tarim River Basin. Their research reveals the ecological evolution patterns of the mainstem, characterizes bank erosion dynamics, and underscores the critical importance of integrated water resource management and ecological conservation. Furthermore, they propose sustainable development recommendations grounded in basin-wide ecological restoration and rational resource utilization. Scholars including Xu Le [17], Li Yuanyuan [18], and Guo Qingchao [19] have conducted systematic analyses of flow characteristics, sediment erosion-deposition patterns, and riverbed deformation across different reaches of the Tarim River Basin using numerical simulation methods. Their work proposes targeted optimization measures for channel regulation and water-sediment dynamics management, providing a scientific basis for river engineering design and adaptive water re-source governance. However, in the field of numerical modeling of fluvial water-sediment dynamics, two-dimensional coupled water-sediment simulations at large spatial scales continue to face the persistent challenge of balancing topographic representation accuracy with computational efficiency. This is particularly pronounced in dynamic modeling of erosion-deposition evolution for long-reach, sediment-laden rivers like the Tarim River’s mainstem, where pronounced spatiotemporal scale coupling effects render local-scale models inadequate for effectively revealing the spatial correlation characteristics of long-reach sediment response patterns. To address this technical challenge, this study employs 30-year (1993–2023) datasets of annual runoff and sediment discharge to conduct two-dimensional water-sediment numerical simulations using MIKE21 software under representative hydrological-sedimentary conditions for the Yingbazha-Wusiman midstream reach of the Tarim River. By incorporating a geo-morphic acceleration factor, we project decadal-scale erosion-deposition evolution trends over the next 10 years. The numerical results systematically analyze potential impacts of channel dynamics on riparian ecosystems and irrigation diversions, thereby providing theoretical foundations for adaptive river management strategies.

## 2. Materials and methods

### 2.1. Study region

**Fig 1:**
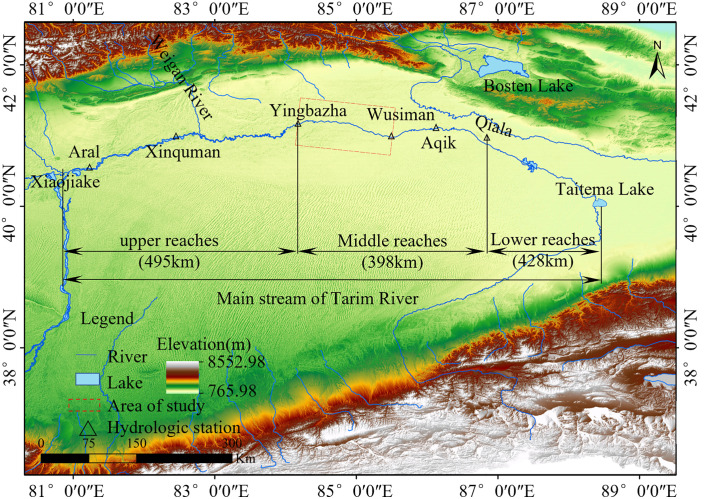
Schematic Diagram of the Research Region. [Image freely available from Open Topography platform (https://portal.opentopography.org/)].

**Fig 2:**
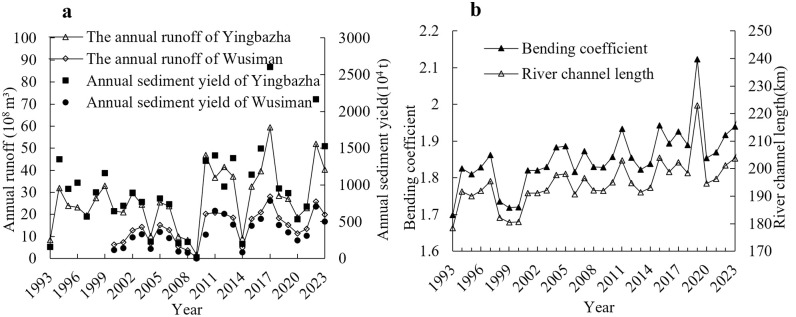
The study of water-sediment conditions and meandering characteristics of river sections. a) Annual changes in runoff and sediment transport in Yingbazha-Wusiman reach from 1993 to 2023, b) Variation of bend coefficient in Yingbazha-Wusiman reach from 1993 to 2023.

The study focuses on the Yingbazha-Wusiman reach of the midstream mainstem of the Tarim River (Fig 1), geographically located between 84°22′-85°12′E and 41°06′-41°23′N. Characterized by sinuosity indices consistently exceeding 1.6, this section represents a quintessential meandering channel system. The study reach exhibits a low gradient of 0.0139%, with water surface widths typically ranging between 200 and 500 meters. The loose soil composition and substantial sediment deposition drive progressive bed aggradation. Coupled with anthropogenic channel excavation practices, these factors have collectively led to the development of numerous anabranching channels within the midstream section [20,21]. The riverbed sediment in this reach is composed predominantly of silty fine sand, with abundant fine-grained sediments and limited medium-sized particles. Both banks predominantly feature oasis ecosystems with dense vegetation, where the soil consists mainly of sandy saline-alkali compositions exhibiting complex mineralogical heterogeneity [22]. Analysis of the 1993–2023 datasets for annual runoff, sediment discharge, channel length, and sinuosity index in the study reach (Fig 2a and b) reveals that annual runoff and sediment discharge exhibit largely consistent trends, with significant interannual fluctuations observed across different hydrological years. Over the past 26 years, 2017 marked the peak water-sediment year, with annual runoff at the Yingbazha Hydrological Station reaching 5.95 × 10^9^ m^3^, while 2009 recorded the minimum water-sediment year, registering a runoff of merely 1.83 × 10^8^ m^3^. The average annual runoff over the years is 2.695 × 10^9^ m^3^, with years having annual runoff between 2 × 10^9^ and 4 × 10^9^ m^3^ accounting for 58%. Comparative analysis of water-sediment influx dynamics and sinuosity indices from 1993 to 2023 reveals that be-tween years exhibiting substantial annual runoff variations, corresponding changes in channel sinuosity demonstrate heightened sensitivity, with morphological adjustments closely coupled to hydrological fluctuations. For instance, in 1993 and 1994, the annual runoff at Yingbazha measured 8.33 × 10^8^ m^3^ and 3.185 × 10^9^ m^3^, with corresponding sinuosity indices of 1.69 and 1.825, respectively; similarly, during 2014 and 2015, annual runoff volumes of 9 × 10^8^ m^3^ and 3.254 × 10^9^ m^3^ coincided with elevated sinuosity indices of 1.83 and 1.94. Furthermore, when annual runoff approaches 3 × 10^9^ m^3^, it tends to induce more pronounced channel migration. For instance, 1994, 2011, and 2019 years with the most significant sinuosity variations in the past three decades registered annual run-off volumes of 3.185 × 10^9^ m^3^, 3.663 × 10^9^ m^3^, and 2.707 × 10^9^ m^3^, respectively. Among these, 2019 exhibited the most pronounced sinuosity variation, showing a 12.3% increase in sinuosity index compared to 2018.

### 2.2. Data sources and preprocessing

The study’s simulation area spans from the upstream Yingbazha Bridge to the downstream Wusiman Junction, encompassing embankments along both riverbanks. The Tarim River Basin incorporates diverse geomorphological features, including high-mountain glaciers, deserts, and oases, exhibiting complex topography and extensive spatial coverage. Constrained by the technical challenges of conventional field surveys and logistical difficulties in deploying observational instrumentation, the mainstem of the Tarim River suffers from a critical deficiency in field-validated topographic datasets. This data scarcity poses significant obstacles to advancing research on channel evolution dynamics within the river’s core reaches [23]. To address this challenge, we systematically screened free and open-source digital elevation model (DEM) datasets covering the entire basin. Following a comprehensive evaluation of data currency, absolute vertical accuracy, horizontal precision, and topographic detail representation, the Copernicus DEM satellite-derived elevation data was selected as the initial topographic input for the hydrodynamic modeling [24]. Since the Copernicus DEM primarily utilizes image data acquired between 2010 and 2015, while only 2018 field-measured data exists for the study reach with no topographic ground truth available for 2010–2015 this study employs the Copernicus DEM as the foundational dataset. Complementary 2018 Landsat optical satellite imagery of the study area was processed using the Normalized Difference Water Index (NDWI) method to extract water bodies, generating hydromorphic masks. These water-body delineations were then applied to clip the preprocessed Digital Elevation Model (DEM), resulting in partitioned elevation datasets: a water-body DEM and a terrestrial DEM. Subsequently, the water-body DEM was rectified using field-measured cross-sections within the study area. Finally, the terrestrial DEM and rectified water-body DEM were mosaicked to generate the finalized Digital Elevation Model [25,26].

### 2.3. Research methodology

#### 2.3.1. Mathematical model.

The hydrodynamic module of MIKE21 is founded on the time-averaged Navier-Stokes equations, where the turbulent shear stress and time-averaged velocity gradient adhere to the Boussinesq approximation and hydrostatic pressure assumption. The governing equations for two-dimensional unsteady shallow water flow are presented in Equations (1)–(3).

Continuity equation:


$$\frac{\partial h}{\partial t}+\frac{\partial h\bar{u}}{\partial x}+\frac{\partial h\bar{v}}{\partial y}=hS$$ (1)

The momentum equation in x direction:


$$\begin{array}{c} \frac{\partial h\bar{u}}{\partial t}+\frac{\partial h{\bar{u}}^{2}}{\partial x}+\frac{\partial h\overset{―}{uv}}{\partial y}=\\ f\bar{v}h-gh\frac{\partial \eta }{\partial x}-\frac{h}{\rho_{0}}\frac{\partial P_{a}}{\partial x}-\frac{gh^{2}}{2\rho_{0}}\frac{\partial p}{\partial x}+\frac{\tau_{sx}}{\rho_{0}}-\frac{\tau_{bx}}{\rho_{0}}-\frac{\text{1}}{\rho_{0}}\left(\frac{\partial s_{xx}}{\partial x}+\frac{\partial s_{xy}}{\partial y}\right)+\frac{\partial \left(hT_{xx}\right)}{\partial x}+\frac{\partial \left(hT_{xy}\right)}{\partial y}+hu_{s}S \end{array}$$ (2)

The momentum equation in y direction:


$$\begin{array}{c} \frac{\partial h\bar{v}}{\partial t}+\frac{\partial h{\bar{v}}^{2}}{\partial x}+\frac{\partial h\overset{―}{uv}}{\partial y}=\\ f\bar{u}h-gh\frac{\partial \eta }{\partial y}-\frac{h}{\rho_{0}}\frac{\partial P_{a}}{\partial y}-\frac{gh^{2}}{2\rho_{0}}\frac{\partial p}{\partial y}+\frac{\tau_{sy}}{\rho_{0}}-\frac{\tau_{by}}{\rho_{0}}-\frac{\text{1}}{\rho_{0}}\left(\frac{\partial s_{yx}}{\partial x}+\frac{\partial s_{yy}}{\partial y}\right)+\frac{\partial \left(hT_{xy}\right)}{\partial x}+\frac{\partial \left(hT_{yy}\right)}{\partial y}+hv_{s}S \end{array}$$ (3)

In the equations: t denotes time; *x* and *y* represent the coordinate directions in the Cartesian coordinate system; *η* is the water level; *h* is the still water depth; *ū* and $\bar{v}$ denote the average velocities in the x and y directions, respectively; *f* is the Coriolis coefficient, where *f*=2*ω*sin*φ*, with *ω* representing the Earth’s angular velocity and *φ* the local latitude; *g* is the gravitational acceleration; *ρ* is the density of water; *ρ₀* is the reference density of water; *P*ₐ** is the local atmospheric pressure; *sₓₓ*, *sₓ*ᵧ**, *s*ᵧ*ₓ*, *s*ᵧᵧ** are the radiation stress components, which are neglected in this study and set to 0; *τ**ₛ*ₓ** and *τ**ₛᵧ*** are the surface wind stress tensors, also neglected and set to 0; *τ*_*bx*_ and *τ*_*by*_ are the bed shear stress tensors; *Tₓₓ*, *Tₓ*_*y*_, *T*_*y*_*ₓ*, *T*_*yy*_ are the horizontal viscous stress terms; *S* is the source term; and *u*ₛ** and *v*ₛ** are the flow velocities of the source term in the x and y directions, respectively.

**Fig 3:**
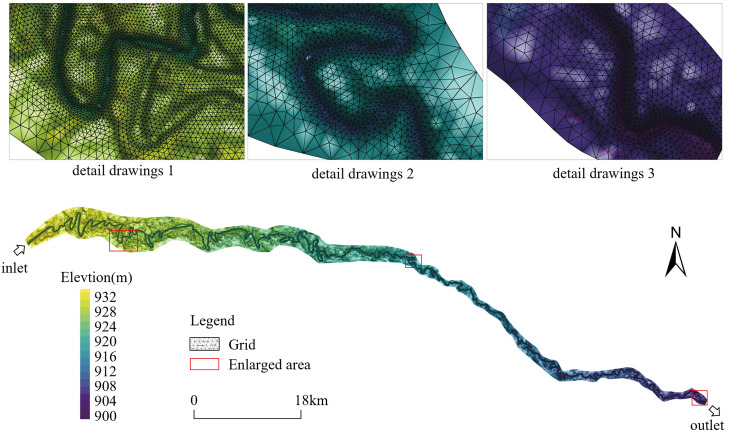
Grid subdivision diagram.

To validate the reliability of the generated Digital Elevation Model (DEM) and ensure its capability to support qualitative analysis of channel evolution and associated ecological impacts, the DEM was rigorously verified against field-measured data from multiple representative cross-sections. The model utilizes a triangular unstructured mesh, with localized refinement implemented in the main channel zone to enhance the simulation accuracy of fluvial morphological evolution. The computational domain comprises 4.55439 × 10^5^ mesh elements and 2.28717 × 10^5^ nodal points, achieving a spatial resolution gradient from 200m (floodplains) to 10 m (channel thalweg). The mesh configuration and refinement strategy are illustrated in Fig 3.

**Fig 4:**
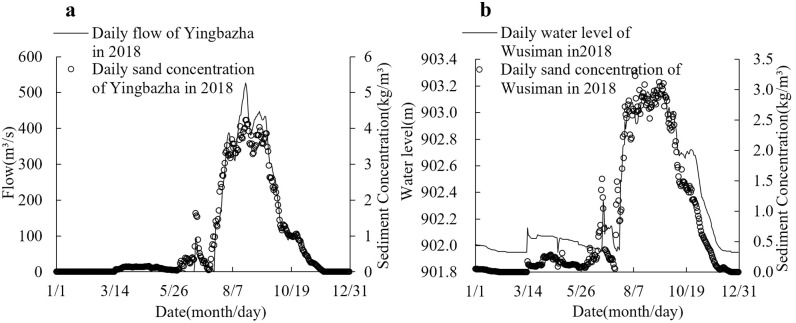
Model inlet and outlet boundary conditions. a) Upstream boundary conditions, b) Downstream boundary conditions.

To ensure simulation accuracy, upstream and downstream hydrometric cross-sections (Yingbazha Bridge and Wusiman Junction) were designated as open boundaries, while both embankment alignments served as closed boundaries. The model adopted a cold-start initialization with initial Manning’s roughness coefficients set to 0.02~0.04 for floodplains and 0.01~0.02 for the main channel. These parameters were subsequently calibrated through iterative simulations. A temporal resolution of 60-second time steps was applied, with the simulation period spanning from January 1 to May 31, 2018. The inlet boundary was defined by the measured discharge-sediment concentration hydrograph from the Yingbazha Hydrological Station (Fig 4a), while the outlet boundary incorporated the observed water level hydrograph and sediment concentration hydrograph at Wusiman (Fig 4b).

#### 2.3.2. Model verification.

Based on the water-sediment inflow conditions at Yingbazha Hydrological Station, the period from June 1 to December 31, 2018, was selected as the model calibration-validation timeframe. The model validation mainly includes water level and sedimentation-erosion cross-section validation. Among them, the sedimentation-erosion validation cross-sections mainly include the following five sections with complete measured topographic data. The specific pile numbers are 592 + 880, 600 + 030, 672 + 325, 673 + 500, and 674 + 000. In addition to using the water level data from pile numbers 600 + 030 and 673 + 500 for validation, the water level validation also selected the river sections where six ecological gates are located, including the Jiefangqu between Yingbazha and Wusiman, Kahatuhedi, Xinshajilike, Patamu, Wushouhan, and Xiadai, for validation. For water level validation, in addition to utilizing 2018 hydrological data from chainages 600 + 030 and 673 + 500, cross-sectional water levels at six ecological sluice gates along the Yingbazha-Wusiman reach Jiefang qu, Kahatuhedi, Xinshajilike, Patamu, Wushouhan and Xiadai were incorporated into the verification framework.

**Fig 5:**
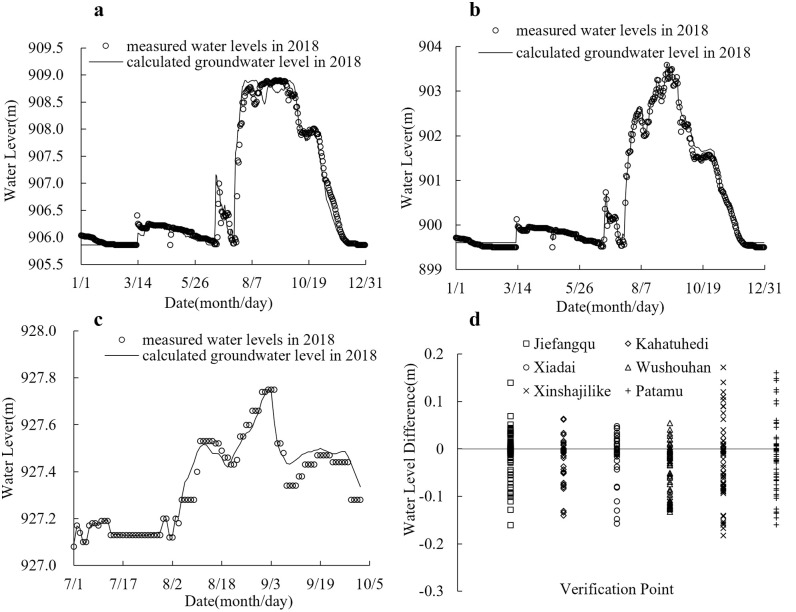
Comparison of calculated water levels with measured data. a) Water Level Verification at Cross-Section 600 + 030, b) Water Level Verification at Cross-Section 673 + 500, c) Water Level Verification at Jiefangqu, d) The residual values between measured and simulated water levels at validation points.

Fig 5a and 5b compare simulated versus measured water levels at cross-sections 592 + 880 and 673 + 500 from January 1 to December 31, 2018. The results demonstrate strong congruence between modeled and observed data, with water level discrepancies constrained within ±0.2 m for most periods. A maximum deviation of 0.48 m occurred during the initial simulation phase, attributable to the model’s cold-start initialization protocol. Fig 5c and 5d present water level validation results at the Jiefangqu ecological sluice gate and residual plots comparing simulated versus observed water levels across all ecological sluice gates. The results demonstrate strong agreement between modeled and measured water level trends, with mean water level discrepancies constrained within ±0.1 m.

**Fig 6:**
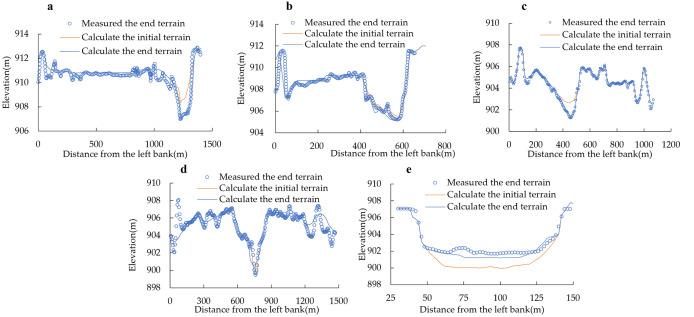
Comparison of the calculated results of erosion and deposition changes at the verification cross-section with measured data Numerical simulation conditions and results analysis. a) Terrain verification of the 592 + 880 section, b) Terrain verification of the 600 + 030 section, c) Terrain verification of the 672 + 325 section, d) Terrain verification of the 672 + 945 section, e) Terrain verification of the 674 + 000 section.

Fig 6a–e shows the comparison between calculated and measured erosion-deposition changes at the five validation cross-sections. All modeled river reaches exhibit varying degrees of scour and deposition within the channel, with the simulated results demonstrating fundamental consistency with the field-measured topography.

## 3. Numerical simulation scenarios with result analysis

### 3.1. Numerical simulation scenarios

In order to explore the impact of different water and sediment conditions on the morphological shaping of the middle reaches of the Tarim River, it is necessary to select a reasonable and representative set of water and sediment series [27]. Over the 30-year period from 1993 to 2023, the maximum annual runoff at Yingbazha Hydrological Station was recorded in 2017, reaching 5.947 × 10^9^ m^3^, while 2009 marked the minimum annual runoff of 1.83 × 10^8^ m^3^. During this period, years with annual run-off below 1.0 billion m^3^ accounted for 12% of the total years, those ranging between 1.0–2.0 × 10^9^ m^3^ constituted 14%, while years with runoff levels of 2.0–3.0 × 10^9^ m^3^ represented 40%. Runoff volumes of 3.0–5.0 × 10^9^ m^3^ occurred in 30% of the years, and exceptional flows exceeding 5.0 × 10^9^ m^3^ were recorded in only 4% of cases. This distribution demonstrates that annual runoff levels most frequently fall within the 2.0–3.0 × 10^9^ m^3^ range.

**Fig 7:**
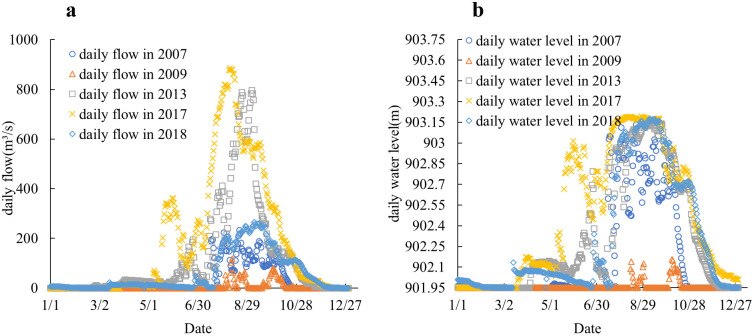
Calculated work condition. a) Inlet boundary condition, b) Outlet boundary condition.

In summary, based on the analysis of the classification of flood years, normal years, and drought years between 1993 and 2023, five different representative years were selected: 2017 (extreme flood year), 2013 (typical flood year), 2018 (typical normal year), 2007 (typical drought year), and 2009 (extreme drought year) [28]. The water and sediment data from these years were used as boundary conditions to simulate the erosion and deposition evolution of the river under different water and sediment conditions. Fig 7a and b shows the boundary conditions used under the operating conditions of different representative years.

In medium-term to long-term morphological dynamic simulations, the Morphological Acceleration Factor (MF), as an effective scale-coupling technique, can effectively bridge the time-scale difference between fluid dynamics and morphological dynamics through a time-scale compression method, thereby significantly improving the computational efficiency of bed evolution [29]. The core principle of this technology is to multiply the bed elevation change by a preset MF value, thereby representing the topographic evolution process over a long time scale. Given that the contribution of short time-scale water and sediment transport processes to the riverbed morphological evolution is relatively limited, to systematically study the impact of different water and sediment conditions on the morphological evolution of the middle reaches of the Tarim River, this study introduces the Morphological Acceleration Factor (MF=10) and conducts numerical simulations of river erosion and deposition evolution under different water and sediment conditions in the river section from Yingbazha to Wusiman River in the middle reaches of the Tarim River.

### 3.2. Result analysis

#### 3.2.1. Scour and fill volume variations.

**Fig 8:**
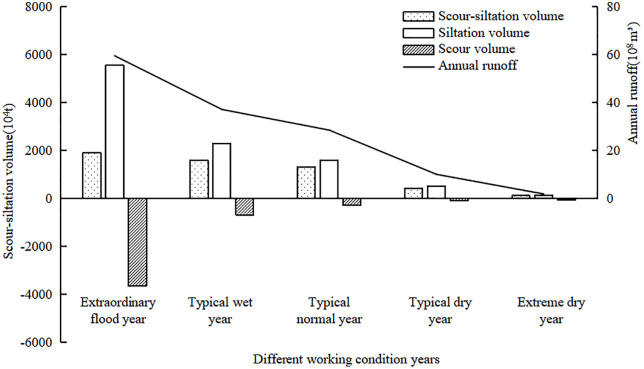
Sedimentation and erosion amounts under various operating conditions.

Fig 8 shows the annual average erosion and deposition volume and the corresponding annual runoff variation characteristics under the influence of different representative water and sediment conditions in the study river section. As can be seen from the figure, during the periods from extreme drought year to extreme flood year, the annual average net sedimentation in the river is significantly correlated with changes in water and sediment conditions. Specifically, the annual average erosion and deposition volume of the river and its proportion to the incoming sediment for each water and sediment condition year are as follows: For the extreme flood year, 1.885 × 10^7^ tons, accounting for 34%; for the typical flood year, 1.592 × 10^7^ tons, accounting for 69%; for the typical normal year, 1.302 × 10^7^ tons, accounting for 82%; for the typical drought year, 4.218 × 10^6^ tons, accounting for 82%; and for the extreme drought year, only 1.109 × 10^6^ tons, accounting for 83%. It is worth noting that, whether in extreme years or typical years, the simulation results of the difference between sedimentation and erosion volumes all show positive values, indicating that the study river section remains in a net sedimentation state, which aligns with the “only sedimentation, no erosion” characteristic commonly observed in inland rivers.

Further analysis of Fig 8 also shows that the unit runoff erosion and deposition intensity is positively correlated with the annual runoff volume. When the runoff volume increases from 1.83 × 10^8^ million m^3^ (extreme drought year) to 5.947 × 10^9^ m^3^ (extreme flood year), the unit runoff sediment transport efficiency (incoming sediment volume/annual runoff volume) significantly rises from 6.06 kg/m^3^ to 9.331 kg/m^3^, an increase of 54%. This growth characteristic may be closely related to the enhanced sediment-carrying capacity of the flow under high flow conditions. Although the simulation results align with the general pattern of “sedimentation dominance” in inland rivers, the study found that when the annual runoff volume exceeds 3.7 × 10^9^ m^3^, the growth rate of erosion volume (+440%) significantly surpasses the growth rate of sedimentation volume (+143%). This phenomenon suggests that the river channel may reach a critical water-sediment condition, triggering a shift in the erosion-deposition mechanism. Specifically, as the incoming flow rate gradually increases, the hydrodynamic conditions undergo a nonlinear surge. This leads to a rapid shift in the sediment transport mode from a “restricted efficiency” state—primarily moving fine particles—to a “high-efficiency transport” state characterized by massive mobilization of coarse grains and a near-saturation concentration of suspended sediment. This transition not only significantly enhances the sediment transport efficiency per unit runoff but also, driven by the intensified flow dynamics, disrupts bed stability by effectively removing the armored layer. Consequently, it triggers severe bed incision and lateral erosion.

#### 3.2.2. Scour and fill depth variations.

**Fig 9:**
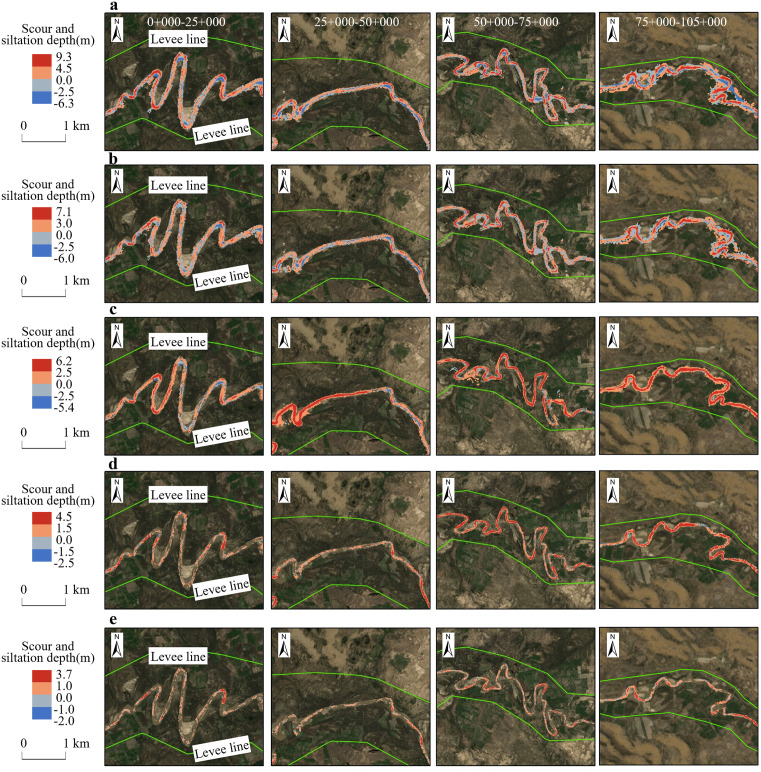
Depth Distribution of Sedimentation and Erosion under Different Working Conditions. [The base map sourced from Landsat (http://landsat.visibleearth.nasa.gov/)]. a) Scour and siltation depth variations in the river reach during extreme flood year, b) Scour and siltation depth variations in the river reach during typical flood year, c) Scour and siltation depth variations in the river reach during typical normal year, d) Scour and siltation depth variations in the river reach during typical drought year, **e)** Scour and siltation depth variations in the river reach during extreme drought year.

Based on the straight-line distance of the river in the study area, the river is di-vided into four sections: 0 + 000–25 + 000, 25 + 000–50 + 000, 50 + 000–75 + 000, and 75 + 000–105 + 000. Fig 9 shows the changes in erosion and sedimentation in some typical segments of these four sections under different operating conditions. Specifically, in the extreme flood year, the maximum erosion and sedimentation depths of the river sections reached 9.3m and −6.3m, respectively, which were the most severe erosion and sedimentation levels among all operating conditions. In contrast, the maxi-mum erosion and sedimentation depths in the extreme drought year were the smallest among all operating conditions, measuring 3.7m and -2m, respectively. In typical flood year, due to the larger flow and higher sediment concentration, the erosion and sedimentation in the river are also more significant. Compared to the extreme flood year, although the depths of erosion and sedimentation are somewhat reduced, the overall range and intensity of erosion and sedimentation remain high, with the maximum erosion and sedimentation depths reaching 7m and -6m, respectively. In typical normal year, the erosion and sedimentation changes in the river are relatively mild, with smaller depths and ranges of erosion and sedimentation. The riverbed morphology remains relatively stable, with the maximum erosion and sedimentation depths reaching 6.2m and −5.4m, respectively. As for a typical drought year, due to the smaller flow, the erosion and sedimentation in the river are further reduced, with limited depths and ranges of erosion and sedimentation. The maximum erosion and sedimentation depths are 4.5m and −2.5m, respectively.

#### 3.2.3. Planform morphological changes induced by scour and fill.

**Fig 10:**
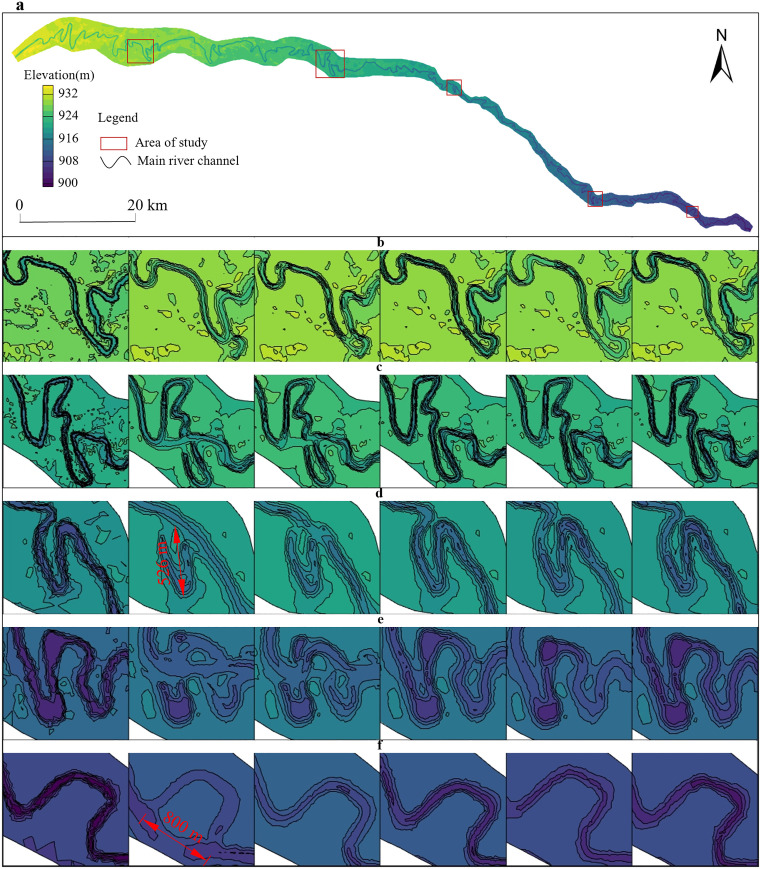
Typical Bending Section River Channel Evolution Map under Different Working Conditions. a) Topographic Map of the River Section, b) Plan View of Scour and Deposition for Typical River Bend Two, c) Plan View of Scour and Deposition for Typical River Bend Three, d) Plan View of Scour and Deposition for Typical River Bend Four, e) Plan View of Scour and Deposition for Typical River Bend Two, f) Plan View of Scour and Deposition for Typical River Bend five. (b),(c),(d),(e) and (f) The figure illustrates the planform evolution of scour and deposition in a typical river bend under different characteristic annual hydrological conditions. From left to right, the panels show: initial topography, followed by channel morphological changes under an extreme flood year, typical high-flow year, typical normal-flow year, typical low-flow year, and extreme drought year conditions.

Fig 10 demonstrates the morphological evolution characteristics of a representative river bend under different hydrological scenarios. As observed, high-flow conditions exert a more dominant influence on channel morphology reshaping, with a significant positive correlation evident between the intensity of channel evolution and the magnitude of flow discharge.

As shown in Fig 10, under flow conditions of typical normal year, typical drought year, and extreme drought year, the morphology of river bend segments exhibits minimal deviation from the original topography. This stability arises from the limited hydrodynamic energy of low-flow regimes, which confines flow activity primarily within the channel boundaries. In contrast, during extreme flood year and typical flood year, the channel undergoes drastic morphological transformations, with marked differences from the initial topography. This is because under high flow conditions, the water flow velocity increases, and its erosion capacity on the riverbanks and riverbed multiplies. Especially in curved river sections, the water flow deviates from its original path due to inertia, directly impacting the convex bank or the weak spots on the concave bank, leading to the cutting of the bank and shoal. For instance, as demonstrated in Fig 10b and e, the meandering reach experienced cutoff events and bank truncation under high-flow conditions of extreme flood year and typical flood year, with maximum lateral migration distances reaching 526 m. In the bend segments shown in Fig 10c and d, prolonged hydro-sedimentary forcing had already significantly narrowed the neck width of the meander loop. Following sustained simulations of high-flow regimes under extreme flood year and typical flood year scenarios, these bends ultimately underwent full meander cutoff and channel straightening. Fig 10f illustrates that under extreme flood year conditions, the river course was forcibly redirected: an originally southeast-northest oriented bend, subjected to direct hydro-dynamic impact at its apex, overflowed into adjacent depressions and scoured a new 800-meter-long channel course. This newly formed channel closely approaches the right-bank levee, posing substantial risks of lateral scour to the embankment infrastructure.

In summary, the analysis reveals that under extreme flood year and typical flood year conditions, meandering reaches frequently experience cutoff events and channel straightening. Conversely, during typical drought year and extreme drought year, these bends exhibit increasingly sinuous patterns. Collectively, the river channel demonstrates a characteristic behavioral pattern: low-flow conditions promote meander development, while high-flow regimes drive channel simplification and straightening.

#### 3.2.4. Thalweg scour and fill variations.

**Fig 11:**
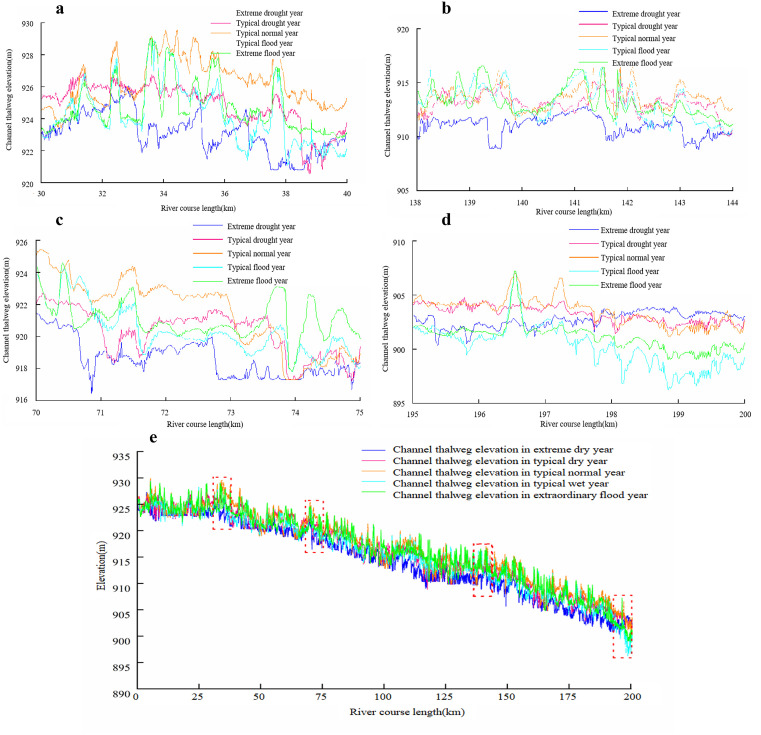
Changes in thalweg elevation of the river channel under different hydrological conditions. a) Interval river section one river deep Hong line, b) Interval river section two river deep Hong line, c) Interval river section three river deep Hong line, d) Interval river section four river deep Hong line, e) Changes in thalweg elevation of the river channel.

Fig 11 illustrates the variation patterns of simulated thalweg lines under different representative year scenarios. The numerical simulation results demonstrate that the evolution trend of thalweg elevation exhibits significant correlation with in-terannual changes in water-sediment conditions. Overall, as the water-sediment flux increases, channel sedimentation intensifies, leading to continuous elevation of the thalweg. However, the scouring-silting mechanism displays nonlinear dynamic responses within specific discharge ranges, which can be characterized by three distinct phases (Fig 11a–e):

(1) Extreme drought year to typical normal year

Within this range, the thalweg elevation generally exhibits a monotonically increasing trend with rising water-sediment discharge, as shown in Table 1. Specifically: For Reach 1 (30 + 000–40 + 000), the average thalweg elevations are 923.09 m, 924.82 m, and 926.28 m, respectively. For Reach 2 (70 + 000–75 + 000), the average thalweg elevations are 918.54 m, 920.26 m, and 921.46 m, respectively. For Reach 3 (138 + 000–144 + 000), the average thalweg elevations are 911.00 m, 913.11 m, and 913.64 m, respectively. For Reach 4 (196 + 000–200 + 000), the average thalweg elevations are 902.74 m, 903.22 m, and 903.44 m, respectively. This is primarily because the sediment-carrying capacity of the flow remains lower than the actual sediment concentration in the channel, leading to continuous sediment deposition.

**Table 1:** Depth distribution of sedimentation and erosion under different working conditions.

River Reach	Deep thalweg elevation in extreme drought year	Deep thalweg elevation in typical drought year	Deep thalweg elevation in typical normal year	Deep thalweg elevation in typical flood year	Deep thalweg elevation in extreme flood year
30 + 000-40 + 000	923.09	924.81	926.28	924.18	924.66
70 + 000-75 + 000	918.54	920.26	921.46	920.27	921.07
138 + 000-144 + 000	911.00	913.11	913.64	913.14	913.23
196 + 000-200 + 000	902.74	903.22	903.44	900.16	901.09

(Unit: m).

(2) Typical normal year to typical flood year

When the flow discharge exceeds a critical threshold, the sediment-carrying capacity of the flow surpasses the actual sediment concentration in the channel, triggering a shift in the scouring-silting mechanism. Consequently, the thalweg elevation transitions from deposition to erosional decline. Specifically: Reach 1 (30 + 000–40 + 000): Average thalweg elevations decreased from 926.28 m to 924.18 m. Reach 2 (70 + 000–75 + 000): Average thalweg elevations declined from 921.46 m to 920.27 m. Reach 3 (138 + 000–144 + 000): Average thalweg elevations reduced from 913.64 m to 913.14 m. Reach 4 (196 + 000–200 + 000): Average thalweg elevations dropped markedly from 903.44 m to 900.16 m, as shown in Table 1. This reversal pattern demonstrates a clear divergence from the previous sedimentation-dominated trend.

(3) Typical flood year to extreme flood year

As water-sediment fluxes surge further, the sediment concentration in the channel once again exceeds the sediment-carrying capacity threshold, causing deposition processes to regain dominance and the thalweg elevation to resume its rising trend. The observed changes are as follows: Reach 1 (30 + 000–40 + 000): Average thalweg elevation increased from 924.18 m to 924.66 m. Reach 2 (70 + 000–75 + 000): Average thalweg elevation rose from 920.27 m to 921.07 m. Reach 3 (138 + 000–144 + 000): Average thalweg elevation increased slightly from 913.14 m to 913.23 m. Reach 4 (196 + 000–200 + 000): Average thalweg elevation rebounded from 900.16 m to 901.09 m, as shown in Table 1.

Overall, the fluvial morphology evolution is predominantly governed by the dynamic equilibrium between the flow’s sediment-carrying capacity and incoming sediment load. The scouring-deposition processes follow a triphase evolutionary pattern of “deposition-erosion-redeposition”, with corresponding thalweg elevation demonstrating a characteristic “rise-fall-rise” phasic fluctuation.

#### 3.2.5. Cross-sectional morphological changes induced by scour and fill.

Furthermore, based on the segmented reaches of the river channel, representative cross-sections with pronounced erosion-deposition dynamics were selected from each of the four designated segments (0 + 000–25 + 000, 25 + 000–50 + 000, 50 + 000–75 + 000, and 75 + 000–105 + 000) for detailed analysis of bed elevation changes.

**Fig 12:**
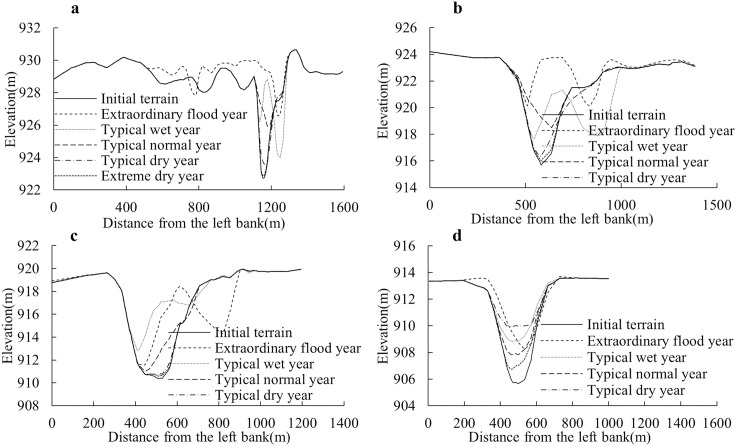
Typical cross-section bed elevation changes. a) Changes in riverbed elevation at typical cross-sections within the 0 + 000–25 + 000 reach under different hydrological conditions, b) Changes in riverbed elevation at typical cross-sections within the 25 + 000–50 + 000 reach under different hydrological conditions, c) Changes in riverbed elevation at typical cross-sections within the 50 + 000–75 + 000 reach under different hydrological conditions, d) Changes in riverbed elevation at typical cross-sections within the 75 + 000–105 + 000 reach under different hydrological conditions.

Fig 12 illustrates the bed elevation changes of each representative cross-section under the hydrological-sedimentary conditions of extreme flood year, typical flood year, typical normal year, typical drought year, and extreme drought year. The analysis reveals that all four cross-sections experienced varying degrees of siltation under different annual flow-sediment regimes. Notably, the volume of bed siltation increases proportionally with rising annual runoff, driving a progressive elevation gain across the riverbed. This siltation-volume variability directly governs the magnitude of bed elevation adjustments. Specifically, as flow discharge increases, the scour effects of the water flow intensify. Under extreme flood year and typical flood year conditions, high discharges drive significant riverbed evolution. In contrast, during typical normal year, typical drought year, and extreme drought year, reduced flow rates result in diminished flow velocities, hindering sediment transport capacity. This leads to siltation of the riverbed and subsequent elevation gain. Consequently, bed elevation changes exhibit a strong correlation with variations in runoff magnitude.

From the cross-sectional elevation changes of the riverbed in Fig 12a, the scour and siltation processes under typical normal year, typical drought year, and extreme drought year conditions primarily occur within the main channel. The most severe sedimentation is observed during typical normal year, where the lowest point of the channel increased from the original elevation of 922.8 m to 925.4 m, representing a rise of 2.6 m. Under the combined effects of water and sediment in typical flood year and extreme flood year, lateral erosion of the main channel began to shift toward the right bank. During typical flood year, the riverbed elevation rose to 924 m. Although the sedimentation was less intense compared to typical normal year, the channel width exhibited expansion. As shown in Fig 12b and c, under the influence of extreme flood year, the typical cross-sections in river reaches 25 + 000–50 + 000 and 50 + 000–75 + 000 both experienced lateral erosion toward the right bank. The degree of river channel sedimentation intensifies with increasing flow discharge. However, as the cross-section location progresses downstream, the response of the main channel’s migration to changes in flow gradually weakens. This phenomenon corresponds well to the trend of the channel’s levee spacing narrowing progressively downstream. In the upper half of the study reach (0 + 000–70 + 000), the levee spacing remains generally between 2~3 km, while in the lower half (70 + 000–105 + 000), it typically narrows to about 1 km. Fig 12d shows that in the river reach 75 + 000–105 + 000, the degree of scour and sedimentation in the typical cross-section follows this order (from highest to lowest): typical drought year, typical flood year, typical normal year, extreme flood year, and extreme drought year. This pattern differs from the previously observed scour-sedimentation trends in other river sections, which may also be related to variations in channel migration magnitude.

Although varying degrees of sedimentation occurred in all four cross-sections during years with higher annual runoff, it can be observed that the channel width increased significantly compared to the original cross-section. This indicates that under high-flow conditions, no significant scouring occurred to lower the riverbed elevation. The likely reason is that bank collapse led to channel widening, reducing flow velocity, while the collapsed sediment deposits entered the main channel, causing sedimentation and consequently raising the riverbed elevation.

## 4. Conclusion

This study utilized the flow discharge, water level, and sediment concentration data from Yingbazha and Wusiman stations as the foundational dataset. Based on validated 2018 topographic data and combined with upstream water-sediment variation patterns, five scenarios were established: extreme flood year, typical flood year, typical normal year, typical drought year, and extreme drought year. By incorporating a geomorphic acceleration factor, the study simulated the river channel’s scour and deposition evolution under different water-sediment conditions over the next decade. The main conclusions are as follows:

(1) The study reach exhibited net sedimentation across all hydrological year types, consistent with the “deposition-dominated” characteristic of inland rivers. Specifically, the sediment budget reached 18.845 million tons during extreme flood year (34% of incoming sediment), while only 1.109 million tons during extreme drought year (83% of incoming sediment). The sediment transport intensity per unit discharge showed a positive correlation with hydrological conditions. When annual runoff exceeded 3.7 × 10^9^ m^3^, the growth rate of scour (+440%) significantly outpaced sedimentation (+143%), suggesting the existence of critical hydro-sedimentary thresholds that may trigger a shift in channel evolution mechanisms.
(2) A systematic analysis was conducted on the evolution patterns of riverbed elevation under varying water-sediment conditions from extreme flood year to extreme drought year. From the perspective of scour-deposition mechanisms, channel morphology evolution is primarily governed by the dynamic equilibrium between sediment transport capacity and incoming sediment load. The scour-deposition process follows a three-phase evolution pattern of “deposition-scour-redeposition”.
(3) During extreme flood year and typical flood year, high discharge rates induce main channel lateral migration and scouring. However, concurrent bank collapse and channel widening paradoxically intensify main channel sedimentation, leading to bed elevation rise. In contrast, under typical normal, typical drought, and extreme drought year with lower discharge, water levels, and flow velocities, scour-deposition processes are confined to the main channel. Notably, the most pronounced main channel sedimentation occurs during typical normal year (e.g., a 2.6 m bed rise in reach 0 + 000–25 + 000). The response differences in different river sections are significant. In the upstream and mid-stream, sedimentation is positively correlated with flow, and the main channel under-goes significant lateral shifting. In contrast, in the downstream section, due to the narrow distance between the embankments, erosion and sedimentation mainly occur within the main river channel.

Based on the aforementioned conclusions, the dynamic balance of river channel erosion and deposition can be maintained through scientific and dynamic water regulation measures, informed by the patterns of erosion and sedimentation under different typical hydrological years revealed in the study. In this process, it is essential to distinguish the different erosion-deposition response mechanisms between the upper-middle reaches and the lower reaches: the upper and middle reaches should focus on accommodating natural processes and reducing adverse sedimentation, while the lower reach must ensure the unobstructed main channel and the safety of embankments. Through this comprehensive management approach characterized by “adapting to natural conditions, implementing zone-specific measures, and dynamic response,” sustainable river management can be achieved more effectively.

## Supporting information

S1 Data Annual changes in runoff and sediment transport in Yingbazha-Wusiman reach from 1993 to 2023. (XLSX)

S2 Data Variation of bend coefficient in Yingbazha-Wusiman reach from 1993 to 2023. (XLSX)

S3 Data Model inlet and outlet boundary conditions. (XLSX)

S4 Data Comparison of calculated water levels with measured data. (XLSX)
